# Single-center experience of transitioning from video-assisted laparoscopic to robotic Heller myotomy with Dor fundoplication for esophageal motility disorders

**DOI:** 10.1186/s12893-023-02202-4

**Published:** 2023-11-10

**Authors:** Xun Jiang, Chunlin Ye, Lei Jiang, Guangxia Wei, Shaohua Dai, Yong Xi, Zhiguo Chen, Bentong Yu, Jian Tang

**Affiliations:** 1 Department of Thoracic Surgery, The First Affiliated Hospital of Nanchang University, Nanchang, 330006 China; 2 National Regional Center for Respiratory Medicine, China Japan Friendship Jiangxi Hospital, Nanchang, 330006 China; 3 Human Genetic Resources Center, The First Affiliated Hospital of Nanchang University, Nanchang, 330006 China

**Keywords:** Heller myotomy, Laparoscopic Heller myotomy with Dor fundoplication, Robotic Heller myotomy with Dor fundoplication, Learning curve

## Abstract

**Background:**

Video-assisted laparoscopic Heller myotomy (LHM) has become the standard treatment option for achalasia. While robotic surgery offering some specific advantages such as better three-dimensional (3D) stereoscopic vision, hand-eye consistency, and flexibility and stability with the endowrist is expected to be shorter in learning curve than that of LHM for surgeons who are proficient in LHM. The aim of this study was to describe a single surgeon’s experience related to the transition from video-assisted laparoscopic to robotic Heller myotomy with Dor fundoplication.

**Methods:**

We conducted a retrospective observational study based on the recorded data of the first 66 Heller myotomy performed with laparoscopic Heller myotomy with Dor fundoplication (LHMD, 26 cases) and with the robotic Heller myotomy with Dor fundoplication (RHMD, 40 cases) by the same surgeon in Department of Thoracic Surgery of The First Affiliated Hospital of Nanchang University in China. The operation time and intraoperative blood loss were analyzed using the cumulative sum (CUSUM) method. Corresponding statistical tests were used to compare outcomes of both serials of cases.

**Results:**

The median operation time was shorter in the RHMD group compared to the LHMD group (130 [IQR 123–141] minutes vs. 163 [IQR 153–169]) minutes, *p* < 0.001). In the RHMD group, one patient (2.5%) experienced mucosal perforation, whereas, in the LHMD group, the incidence of this complication was significantly higher at 19.2% (5 patients) (*p* = 0.031). Based on cumulative sum analyses, operation time decreased starting with case 20 in the LHMD group and with case 18 in the RHMD group. Intraoperative blood loss tended to decline starting with case 19 in the LHMD group and with case 16 in the RHMD group.

**Conclusions:**

Both RHMD and LHMD are effective surgical procedures for symptom relief of achalasia patients. RHMD demonstrates superior outcomes in terms of operation time and mucosal perforation during surgery compared to LHMD. Proficiency with RHMD can be achieved after approximately 16–18 cases, while that of LHMD can be obtained after around 19–20 cases.

**Supplementary Information:**

The online version contains supplementary material available at 10.1186/s12893-023-02202-4.

## Introduction

Achalasia is the most prevalent primary motility disorder of the esophagus. It is characterized by esophageal smooth muscle motility disorder and impaired lower esophageal sphincter (LES) relaxation in response to swallowing [1]. The common symptoms of achalasia include dysphagia, regurgitation, heartburn, chest pain, weight loss, and respiratory complications such as nocturnal cough, aspiration, and pneumonia [2–6].

The minimally invasive Heller myotomy with partial fundoplication is the gold-standard surgical treatment recommended by the American College of Gastroenterology (ACG) for patients with achalasia [5]. While laparoscopic Heller myotomy has been widely utilized and has demonstrated outstanding clinical outcomes [7–10], the robot-assisted Heller myotomy is emerging as a promising candidate for treating achalasia. The da Vinci Si/Xi robotic surgical system has advantages in providing better three-dimensional (3D) stereoscopic vision, hand-eye consistency, and flexibility and stability with the endowrist. However, this sophisticated system can only be operated by the surgeon after specific training [11–13]. Studies demonstrating the learning curve of this technique remain scarce.

The current study aims to compare the learning curve of the video-assisted laparoscopic Heller myotomy with Dor fundoplication (LHMD) to the learning curve of the robotic Heller myotomy with Dor fundoplication (RHMD) from the same experienced surgeon for treating achalasia and the outcomes of both serials of cases. We also share the experience of the transition from LHMD to RHMD. We hypothesized that the learning curve for RHMD should be flatter in a surgeon who is already proficient in LHMD.

## Methods

### Study design

This single-center retrospective study was conducted at the Department of Thoracic Surgery of The First Affiliated Hospital of Nanchang University in China complied strictly to the revised Declaration of Helsinki. Ethical approval was obtained from the ethics committee of The First Affiliated Hospital of Nanchang University (approval number: 2,021,042). Between November 2013 and March 2021, 66 patients (≥ 18 years) diagnosed with achalasia underwent primary minimally invasive Heller myotomy with Dor fundoplication at our center were recruited in this study. Inform consents were obtained from all participants. Diagnosis of achalasia was based on the patient’s symptoms, barium esophagram, upper endoscopy, and manometry. The achalasia subtypes were classified by applying high-resolution manometry (HRM) according to the Chicago classification [14, 15]. All participants were asked to complete a questionnaire that includes items of the Eckardt symptom score, Stooler score, and Gastrointestinal Quality of Life Index (GIQLI) score [16–18].

### Professional profile of the reflecting surgeon

Both RHMD and LHMD for the enrolled patients were performed by the same surgeon who has more than 20 years of surgical practice and a high level of experience in the clinical treatment of achalasia. The surgeon had performed more than 1000 video-assisted thoracoscopic surgeries and 50 robot-assisted thoracoscopic surgeries before the start of performing the RHMD.

### The adoption of RHMD verse LHMD

In 2013, the study department began performing LHMD for all patients diagnosed with achalasia admitted to this center. This continued until September 2016, when a robotic surgical system was introduced. Subsequently, RHMD became the preferred approach, unless the patient declined robotic surgery.

### Procedures of the RHMD and LHMD

#### RHMD approach

**Fig. 1:**
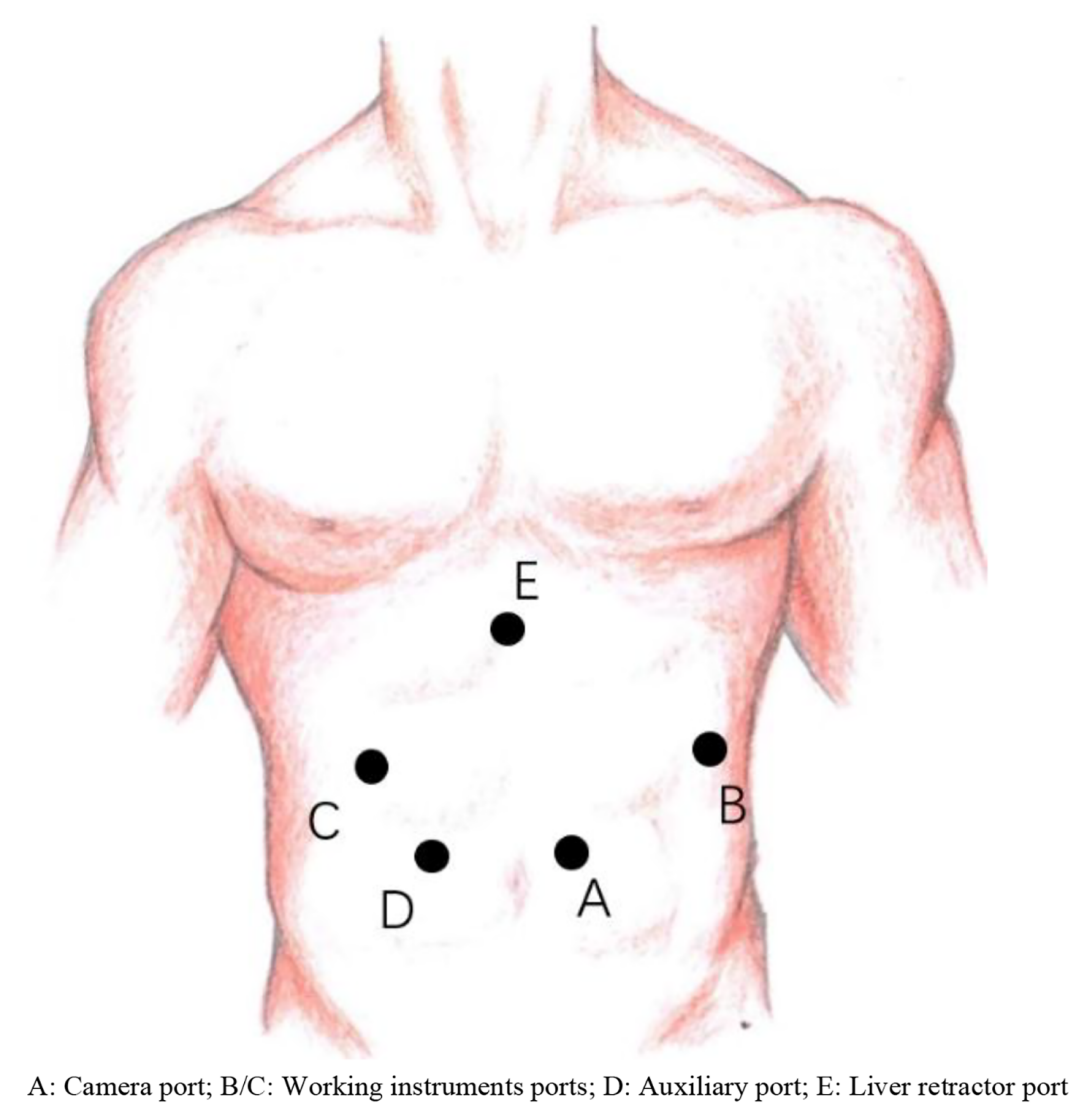
Trocar position for RHMD **A:** Camera port; **B/C:** Working instruments ports; **D:** Auxiliary port; **E:** Liver retractor port

The RHMD approach used the da Vinci Si/Xi surgical system (Intuitive Surgical Inc., Sunnyvale, CA, USA). Patients underwent general anesthesia with single-lumen endotracheal intubation and were positioned supine with a high head and low slope of the feet (approximately 20 degrees) and an 8–10 degrees tilt to the right. The da Vinci Xi system applied a trocar of 8 mm diameter, while the camera port required a 12 mm trocar in the da Vinci Si surgical system. All auxiliary ports used 12 mm trocars. Five ports were placed during the robotic Heller myotomy (RHMD) procedure(Both SI and XI systems are consistent). The camera port was positioned 1–2 cm to the left of the navel, while the two working instrument ports were placed at the lower margin of the left and right costal arch, between the midclavicular line and the anterior axillary line. An auxiliary port was placed 3–4 cm to the left of the camera port, and a liver retractor port was placed under the xiphoid process to provide optimal visualization and instrument access during the RHMD procedure (Fig. 1).

We utilized several energy devices as part of the robotic system. These included a harmonic curved shear, a permanent cautery hook, a fenestrated bipolar forceps, and a large needle. Pneumoperitoneum pressure was set at 12 mmHg to facilitate access to the surgical site. Once the instruments were properly positioned, the second assistant inserted a liver retractor through the liver retractor port and pulled the left lobe of the liver to the right front to expose the cardia, abdominal esophagus, and stomach fundus. The adipose tissue at the esophagogastric junction was removed using the harmonic curved shear to fully expose the anterior esophageal wall and esophageal hiatus, taking care to protect the anterior wall vagus nerve branch.

**Fig. 2:**
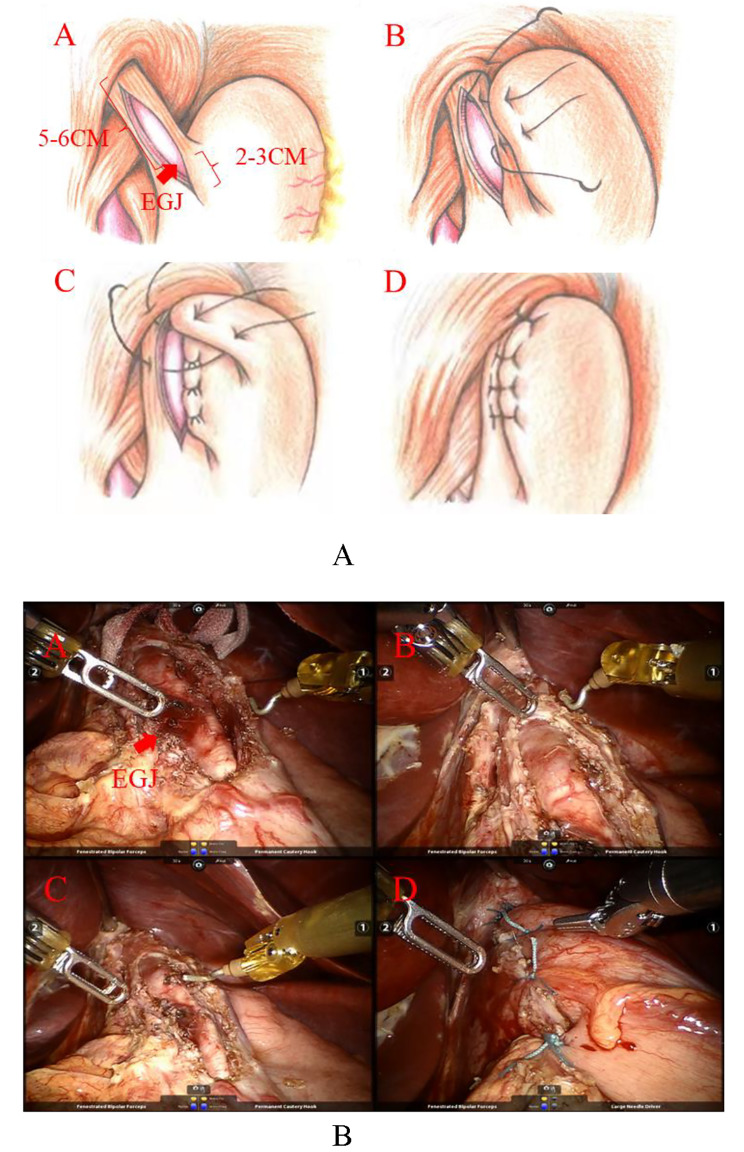
Details of Heller Myotomy Details of Heller Myotomy with Dor fundoplication (Hand drawing). **A:** The length of the myotomy was approximately 5–6 cm upwards and at least 2 cm downwards based on the EGJ; **B-D:** The left and right margins of the esophageal muscle layer and the anterior wall of the fundus of the stomach were then intermittently sutured together Details of Heller Myotomy with Dor fundoplication (Actual operation). **A-C:** The length of the myotomy was approximately 5–6 cm upwards and at least 2 cm downwards based on the EGJ; **D**: The left and right margins of the esophageal muscle layer and the anterior wall of the fundus of the stomach were then intermittently sutured together

After dissecting the esophagus, a cotton rope was used to wrap around it to enhance traction, fully exposing the lower esophagus and bilateral diaphragmatic crus. Gastroscopy was performed to determine the stenosis (esophagogastric junction [EGJ]) under the guidance of gastroscopy. We applied a permanent cautery hook to longitudinally divide the longitudinal muscle along the anterior wall of the esophagus at the EGJ, followed by the underlying circular fibers until it reached the mucosal layer. The length of the myotomy was approximately 5–6 cm upwards and at least 2 cm downwards based on the EGJ. After the myotomy, the outward distended esophageal mucosa was visible through the incision. The edges of the incision were lifted, and we used a permanent cautery hook to divide the submucosa with a range of 1/2 ~ 2/3 of the circumference of the esophagus. Gastroscopy was then performed to check for any mucosal perforation or stenosis, and a gastric tube was inserted. Any small mucosal perforation was repaired using absorbable interrupted 3 − 0 sutures, and we also employed gastroscopy again to confirm that there was no esophageal mucosal leakage. Subsequently, an anterior Dor (180-degree) fundoplication was performed by turning the fundus of the stomach over to the lesser curvature of the stomach to cover the incision of the anterior wall of the esophagus. The left and right margins of the esophageal muscle layer and the anterior wall of the fundus of the stomach were then intermittently sutured together. Finally, we placed a drain in the lesser curvature of the stomach (Fig. 2).

#### LHMD approach

The trocar positions and steps of operation for LHMD were similar to the RHMD approach above but without the manipulation of the robotic arms-endowrist.

### Statistical analyses

The statistical analyses were conducted using IBM SPSS Statistics Version 26.0 (IBM Corp., Armonk, NY, USA). For categorical variables, comparisons between the two groups were made using either Pearson’s Chi-squared test or Fisher’s exact test. Continuous variables that were normally distributed were presented as mean ± standard deviation (SD) and compared using Student’s t-test. Non-normally distributed continuous variables were presented as median (interquartile range [IQR]) and compared using either the Mann-Whitney U test or the Wilcoxon rank-sum test, depending on the nature of the non-compliance. A significance level of α = 0.05 (two-tailed) was used, and differences with *p* < 0.05 were considered statistically significant.

The operation time and intraoperative blood loss were analyzed using the cumulative sum (CUSUM) method, which involves calculating the running total of differences between individual data points and the mean of all data points [19, 20].

### Follow-up evaluation

We followed up with the recruited patients after the day of surgery for at least one year. Postoperative outpatient follow-up procedures, including physical examination, barium esophagram, upper endoscopy, 24-hour pH monitoring, and high-resolution manometry, were performed at 3, 6, and 12 months after surgery. Subsequently, patients had the option of regular online follow-up. A questionnaire survey was conducted on patients within the first year after surgery, encompassing items of Eckardt symptom score, Stooler score, GIQLI score, postoperative symptoms, and reintervention status.

## Results

### Baseline characteristics

The current study enrolled 66 patients with achalasia who had undergone minimally invasive Heller myotomy with Dor fundoplication between November 2013 and March 2021. Of them, 40 (61%) patients with RHMD and 26 (39%) with LHMD, respectively. Table 1 depicts the baseline characteristics of the recruited patients. Type II achalasia was the most common achalasia subtype (55.0% in the RHMD group and 53.8% in the LHMD group). Dysphagia (95.0% in the RHMD group and 92.3% in the LHMD group) was the most common preoperative symptom. 15 patients in the RHMD group (6 for Drug therapy, 7 for PD, and 2 for POEM) and 10 patients in the RHMD group (3 for Drug therapy and 7 for PD) received preoperative treatment. A total of 58 patients (35/40, 23.63 ± 4.17 mmHg in RHMD group and 23/26, 23.65 ± 3.85 mmHg in LHMD group, *p* = 0.958) completed preoperative HRM for integrated relaxation pressure (IRP). There were no significant differences between patients of two groups in their age, gender, body mass index (BMI), achalasia subtype, preoperative symptoms, and preoperative treatment.

**Table 1:** Baseline characteristics of the study patients

	RHMD (N = 40)	LHMD (N = 26)	P-value
Age (years), mean ± SD	43.10 ± 18.99	44.84 ± 11.46	0.643
Gender, n (%)			0.482
Male	18 (45.0%)	14 (53.8%)	
Female	22 (55.0%)	12 (46.2%)	
BMI (kg/m2), mean ± SD	20.81 ± 3.56	22.09 ± 2.60	0.096
Achalasia subtype, n (%)			1.000
I	10 (25.0%)	7 (26.9%)	
II	22 (55.0%)	14 (53.8%)	
III	3 (7.5%)	2 (7.7%)	
Unknown	5 (12.5%)	3 (11.5%)	
Preoperative symptoms, n (%)			
Dysphagia	38 (95.0%)	24 (92.3%)	0.644
Regurgitation	24 (60.0%)	15 (57.7%)	0.852
Heartburn	25 (62.5%)	14 (53.8%)	0.485
Chest pain	8 (20.0%)	6 (23.1%)	0.765
Weight loss	3 (7.5%)	6 (23.1%)	0.138
Respiratory symptoms	6 (15.0%)	3 (11.5%)	1.000
Vomiting	13 (32.5%)	6 (23.1%)	0.409
Preoperative treatment, n (%)			
Drug therapy	6 (15.0%)	3 (11.5%)	1.000
PD	7 (17.5%)	7 (26.9%)	0.360
POEM	2 (5.0%)	0 (0.0%)	0.515

BMI: body mass index; SD: standard deviations; RAHM:robotic Heller myotomy and Dor fundoplication; LHMD: video-assisted laparoscopic Heller myotomy and Dor fundoplication; PD: pneumatic dilation; POEM: per-oral endoscopic myotomy

### Surgical quality and follow-up outcomes

Indicators of the surgical process and follow-up outcomes in RHMD and LHMD groups were summarized (Table 2). No perioperative deaths were observed in both groups. The median operation time was significantly shorter in the RHMD group compared to the LHMD group (130 [IQR 123–141] minutes vs. 163 [IQR 153–169]) minutes, *p* < 0.001). Despite no statistically significant difference, the median intraoperative blood loss was 42 (IQR 27–55) ml in RHMD, which was lesser than 51 (IQR 39–60) ml in the LHMD group (*p* = 0.135).

**Table 2:** Summary of perioperative and follow-up outcomes

	RHMD (N = 40)	LHMD (N = 26)	P-value
Operation time (min), median (IQR)	130 (123–141)	163 (153–169)	＜0.001
Blood loss (ml), median (IQR)	42 (27–55)	51 (39–60)	0.135
Mucosal perforation, n (%)	1 (2.5%)	5 (19.2%)	0.031
Conversion to open, n (%)	0 (0.0%)	1 (3.8%)	0.394
Postoperative relief, n (%)	39 (97.5%)	25 (96.2%)	1.000
Postoperative symptoms, n (%)			
Dysphagia	4 (10.0%)	5 (19.2%)	0.301
Regurgitation	8 (20.0%)	9 (34.6%)	0.185
Heartburn	2 (5.0%)	1 (3.8%)	1.000
Chest pain	0 (0.0%)	1 (3.8%)	0.394
Reintervention, n (%)			
Drug therapy	20 (50.0%)	18 (69.2%)	0.122
PD	4 (10.0%)	3 (11.5%)	1.000
Heller myotomy	2 (5.0%)	1 (3.8%)	1.000
Eckardt symptom score, n (%)			0.557
≤ 3	39 (97.5%)	24 (92.3%)	
> 3;	1 (2.5%)	2 (7.7%)	

IQR: interquartile range; RAHM:robotic Heller myotomy and Dor fundoplication;LHMD: video-assisted laparoscopic Heller myotomy and Dor fundoplication; PD: pneumatic dilation; Drug therapy: using calcium channel blockers or proton pump inhibitors

In the RHMD group, one patient (2.5%) experienced mucosal perforation, whereas, in the LHMD group, the incidence of this complication was significantly higher at 19.2% (5 patients) (*p* = 0.031). The most frequently affected site of mucosal perforation was the (EGJ). No conversions to open surgery occurred in the RHMD group, whereas one patient in the LHMD group required conversion due to severe peritoneal adhesions. In terms of postoperative symptom relief, 97.5% (39/40) of patients in the RHMD group and 96.2% (25/26) in the LHMD group reported short-term subjective palliation of symptoms. At the one-year follow-up, 97.5% (39/40) of patients in the RHMD group and 96.2% (25/26) in the LHMD group achieved an Eckardt score of ≤ 3, indicating successful long-term symptom control.

The median duration of follow-up was 35 (17–52) months in the RHMD group and 57 (45–72) months in the LHMD group. During the first year of follow-up, postoperative symptoms were reported in 10 patients (25.0%) in the RHMD group and 9 patients (34.6%) in the LHMD group. Among these patients, regurgitation occurred in 8 patients (20%) in the RHMD group and 9 patients (34.6%) in the LHMD group. However, the difference between the two groups was not statistically significant (*p* = 0.185). Regarding reintervention, 23 patients (57.5%) in the RHMD group (20 for drug therapy, 4 for POEM, and 2 for Heller myotomy) and 18 patients (69.2%) in the LHMD group (18 for drug therapy, 3 for POEM, and 1 for Heller myotomy) required postoperative treatment. No statistically significant differences were observed between the two groups in terms of postoperative symptoms and reintervention.

### Comparison of questionnaire scores

Table 3 displays the comparison of Eckardt symptom score, Stooler score, and GIQLI score before and after surgery for the RHMD and LHMD groups, respectively. In both groups, the above-mentioned scores significantly improved one year after surgery compared to preoperation (*p* < 0.001). Table 4 summarizes the comparison of pre-and post-operative scores between the RHMD and LHMD groups. There were no significant differences in postoperative Eckardt symptom scores (0.80 ± 0.88 in RHMD group vs. 0.92 ± 0.98 in LHMD group, *p* = 0.597), Stooler scores (0.13 ± 0.40 in RHMD group vs. 0.23 ± 0.51 in LHMD group, *p* = 0.355), and GIQLI scores (120.85 ± 8.37 in RHMD group vs. 117.73 ± 7.12 in LHMD group, *p* = 0.122) between the two groups. Similarly, there were no significant differences in the preoperative scores between the two groups.

**Table 3:** Preoperation and postoperation scores comparison

RHMD group	Preoperation	Postoperation	P-value
Eckardt, mean ± SD	8.05 ± 1.47	0.80 ± 0.88	＜0.001
Stooler, mean ± SD	2.23 ± 0.73	0.13 ± 0.40	＜0.001
GIQLI, mean ± SD	85.93 ± 7.19	120.85 ± 8.37	＜0.001
LHMD group	Preoperation	Postoperation	P-value
Eckardt, mean ± SD	7.62 ± 2.02	0.92 ± 0.98	＜0.001
Stooler, mean ± SD	2.42 ± 0.90	0.23 ± 0.51	＜0.001
GIQLI, mean ± SD	86.19 ± 6.24	117.73 ± 7.12	＜0.001

**Table 4:** Scores comparison between RHMD group and LHMD group

Preoperation	RHMD (N = 40)	LHMD (N = 26)	P-value
Eckardt, mean ± SD	8.05 ± 1.47	7.62 ± 2.02	0.349
Stool, mean ± SD	2.23 ± 0.73	2.42 ± 0.90	0.331
GIQLI, mean ± SD	85.93 ± 7.19	86.19 ± 6.24	0.889
Postoperation	RHMD (N = 40)	LHMD (N = 26)	P-value
Eckardt, mean ± SD	0.80 ± 0.88	0.92 ± 0.98	0.597
Stool, mean ± SD	0.13 ± 0.40	0.23 ± 0.51	0.355
GIQLI, mean ± SD	120.85 ± 8.37	117.73 ± 7.12	0.122

### Cumulative sum analysis

**Fig. 3:**
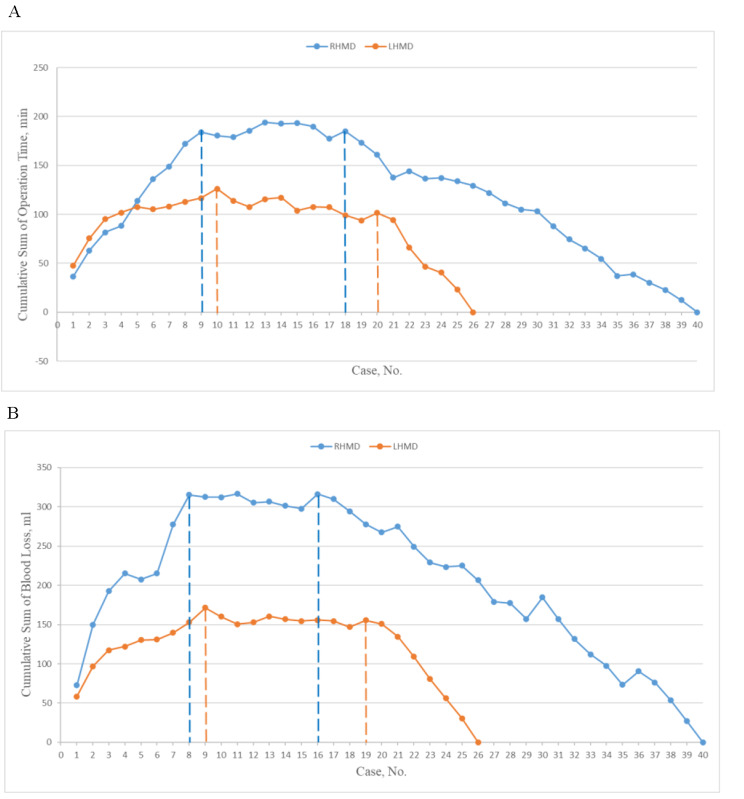
Learning curves of RHMD and LHMD

The learning curves of RHMD and LHMD reflected by operation time and intraoperative blood loss, according to cumulative sum analysis, are shown in Fig. 3. The CUSUM charts are divided into three phases to indicate the level of mastery achieved. For operation time, phase 1 represents the initial learning stage comprising the first nine cases of the RHMD group and the first 10 cases of the LHMD group. Phase 2 represents the consolidation stage spanning the 10th to the 18th cases of the RHMD group and the 11th to the 20th cases of the LHMD group, signifying the surgeon’s increased proficiency. Phase 3 represents the experienced stage (plateau), starting from case 19 in the RHMD group and case 21 in the LHMD group, indicating the achieved proficiency of the surgeon.

Similar patterns were seen in the intraoperative blood loss. Phase 1 included the first eight cases of the RHMD group and the first nine cases of the LHMD group. In phase 2, the RHMD group included cases from the 9th to the 16th, and the LHMD group included cases from the 10th to the 19th. Phase 3 began with case 17 in the RHMD group and case 20 in the LHMD group.

## Discussion

In this single-center retrospective study, we compared the effectiveness of two different surgical approaches- RHMD and LHMD in patients diagnosed with achalasia. The study also evaluated the learning curves of both approaches in the same surgeon, transitioning from LHMD to RHMD. Our results showed that both the RHMD and LHMD groups achieved significant symptom relief after the procedures. However, the RHMD approach had a statistically shorter operation time and a lower rate of mucosal perforation compared to LHMD. Proficiency with RHMD was reached after cases 16–18, while LHMD proficiency was obtained after cases 19–20.

Our study found that both the RHMD and LHMD approaches were effective in relieving the symptoms of patients with achalasia. Of the 66 patients included in the study, 97.5% in the RHMD group (39/40) and 96.2% in the LHMD group (25/26) experienced short-term symptom relief subjectively. At 1-year follow-up, 97.5% of the patients in the RHMD group (39/40) and 92.3% in the LHMD group (24/26) had Eckardt scores of ≤ 3 points, which is consistent with findings in previous studies [11–13, 21–23].

Raja and colleagues [13] conducted a retrospective study comparing RHMD and LHMD approaches for achalasia treatment. The study used both subjective (symptom relief) and objective (esophageal emptying) measures to assess treatment effects. In another study, Werner and colleagues [18] evaluated the improvement of esophageal function by measuring the IRP of LES from baseline to 24 months in the comparison between LHMD and POEM. However, our study did not use objective indicators to assess treatment effects due to various reasons [24]. Nevertheless, it is important to note that the primary goal of any achalasia treatment is to provide relief of symptoms, leading to an improved quality of life, rather than the eradication of the disease. While inconsistencies between subjective and objective results have been reported in previous studies [13, 25–27], achieving patient symptom relief is still the most desirable outcome of treatment.

In terms of perioperative outcomes, our study suggested the operation times in the RHMD group were statistically shorter than that in the LHMD group (130 [123–141] min) vs. 163 [153–169] min, *p* < 0.001). Several studies were showing shorter operation times in the RHMD group without statistical inference [13, 28], while few studies have reported the advantages of RHMD in operation time statistically and some studies even show longer operation time in the RHMD group. The differences above may result from the learning curve stage of surgeons, the technical advantages of a robotic surgical system, and surgeons’ previous experience in LHMD.

Based on published data, the rates of intraoperative mucosal perforation during LHMD in various studies range from 1.8 to 16.0% [10–13, 21, 22, 28–31], whereas the rates of RHMD range from 0.0 to 2.7%. Our study found that the rate of intraoperative mucosal perforation in the RHMD group was statistically lower than that in the LHMD group (2.5% vs. 19.2%, *p* = 0.031), which is consistent with previous research [11, 12, 21, 22, 28, 30, 31]. In our study, intraoperative mucosal perforation frequently occurred at the EGJ, where it was particularly challenging for laparoscopic procedures to create the necessary submucosal plane to divide the muscle fibers, which changed direction from circular in the esophagus to oblique in the stomach [12]. The robotic surgical system’s superior visualization and finer motor control may help surgeons overcome this difficulty.

Normative questionnaires are useful tools for assessing symptom severity, response to treatment, and quality of life in patients with achalasia. The Eckardt symptom score is a widely used 4-item self-report scale that assesses the most common achalasia symptoms, including dysphagia, regurgitation, chest pain, and weight loss, using a 0–3-point scale for each item, with a maximum score of 12 points [16]. This instrument has demonstrated fair reliability and validity, and a post-treatment Eckardt symptom score of ≤ 3 points is widely considered to indicate excellent efficacy of achalasia management [32, 33].

In this study, we evaluated the treatment effects and quality of life of patients with achalasia using three different scales: the Eckardt symptom score, the Stooler score, and the Gastrointestinal Quality of Life Index (GIQLI). The results showed significant improvements in these scores for the whole cohort from pre-operation to post-operation, indicating that both RHMD and LHMD effectively relieve achalasia symptoms. There were no significant differences in pre-operation and post-operation scores between the two groups and the percentage of patients with Eckardt scores of ≤ 3 points at 1 year were similar in both groups, with ≥ 97% in the RHMD group and ≥ 92% in the LHMD group (*p* = 0.557). However, it should be noted that although the difference was not statistically significant, patients in the RHMD group had a trend toward a better quality of life compared to those in the LHMD group. Huffman and colleagues [30] reported that the robotic surgical system was a more precise and safer approach than laparoscopic myotomy, leading to better quality-of-life indices postoperatively. Similarly, Raja and colleagues [13] found that patients in the RHMD group had improved esophageal emptying, improved symptom palliation, and decreased risk of reintervention compared to those in the LHMD group.

The learning curve is a commonly used term to describe the time and experience required to become proficient in a new skill or procedure. In the context of surgery, the learning curve specifically refers to the number of cases needed to achieve a level of proficiency in performing a particular procedure [34]. The evaluation of learning curves by tracking a single variable such as operation time were applied in previous studies. In our study, we utilized the CUSUM method, which is independent of sample size and is capable of detecting small but continuous shifts in data, to evaluate the learning curve of both RHMD and LHMD. Our results showed that proficiency in RHMD was achieved after approximately 16–18 cases, while proficiency in LHMD was achieved after approximately 19–20 cases. Lim and colleagues [35] previously reported that 18 cases were required to achieve mastery in robotic Heller myotomy (RHM) with selective fundoplication, based on operation time. Our findings are also consistent with previous studies reporting a learning curve of 16 to 20 cases for LHMD [36, 37].

An important factor that can influence the learning curve for RHMD is the surgeon’s prior experience in LHMD. Conversely, Baldonado and colleagues [38] found no definitive learning curve for robotic lobectomy in surgeons experienced in video-assisted thoracoscopic surgery (VATS). Gómez-Hernández and colleagues [20] reported that individual surgeons experienced in VATS and open surgery still need to go through a new learning curve when adapting to robotic surgery. On the other hand, Chao and colleagues [39] reported that a surgeon with prior experience in VATS could shorten the learning curve for robotic surgery. It remains controversial whether previous experience in thoracoscopic or laparoscopic surgery affects the learning curve for robotic surgery. Although the learning curve for RHMD is influenced by various factors such as a surgeon’s motivation for progression in robotic surgery, baseline education, the inherent complexity of the surgery, and other variables [34], the RHMD learning curve appeared to decrease with the experienced laparoscopic surgeon.

### Limitations

Several limitations remain in this study. Firstly, given the rarity of achalasia and the availability of alternative non-surgical treatments, this retrospective, single-center study was based on a small sample size, which made it difficult to perform propensity score matching to reduce confounding effects. Secondly, not all patients completed preoperative high-resolution manometry for integrated relaxation pressure (IRP), and we did not use objective indicators to assess treatment effects due to practical constraints. Lastly, this study only analyzed operation time and intraoperative blood loss to describe the learning curve. Since learning robotic surgery is a complex, multifactorial process, analyses should include additional measures such as the rate of conversion to open surgery, number of intraoperative complications, ICU admission, postoperative complications, and so on.

## Conclusion

In conclusion, both RHMD and LHMD are effective surgical procedures for symptom relief of achalasia patients. RHMD demonstrates superior outcomes in terms of operation time and mucosal perforation during surgery compared to LHMD. Proficiency with RHMD can be achieved after approximately 16–18 cases, while that of LHMD can be obtained after around 19–20 cases.

### Electronic supplementary material

Below is the link to the electronic supplementary material.

Supplementary Material 1

## Data Availability

The datasets generated and/or analysed during the current study are not publicly available due the hospital’s policy and the consideration privacy of the participants but are available from the corresponding author on reasonable request. This study was conducted strictly complied with the revised Declaration of Helsinki.
