# Supplementary material for: Single-center experience of transitioning from video-assisted laparoscopic to robotic Heller myotomy with Dor fundoplication for esophageal motility disorders
Source: BMC Surg. 2023 Nov 10;23:341. doi: 10.1186/s12893-023-02202-4 (PMC10638721; doi:10.1186/s12893-023-02202-4)
Supplement: Supplementary file 1 — Supplementary Material 1 [file 12893_2023_2202_MOESM1_ESM.docx]

Supplementary material

**Table S1** Eckardt score

| Symptom | Score | | | |
| --- | --- | --- | --- | --- |
|  | 0 | 1 | 2 | 3 |
| Dysphagia | None | Occasional | Daily | Every meal |
| Regurgitation | None | Occasional | Daily | Every meal |
| Chest pain | None | Occasional | Daily | Every meal |
| Weight loss (kg) | None | <5 | 5–10 | >10 |

Score are added up to generate a total score between 1 and 12.

**Table S2** Stooler score

| Stooler score | |
| --- | --- |
| Grade 0 | can eat normal food |
| Grade 1 | obstruction in the consumption of soft food |
| Grade 2 | can consume a semi-liquid diet |
| Grade 3 | can consume a liquid diet |
| Grade 4 | difficulty or inability in ingesting liquids |

**Table S3** GIQLI score

| GIQLI score |
| --- |
| 1. How often during the past 2 weeks have you had pain in the abdomen?   all of the time, most of the time, some of the time, a little of the time, never |
| 1. How often during the past 2 weeks have you had a feeling of fullness in the upper abdomen?   all of the time, most of the time, some of the time, a little of the time, never |
| 1. How often during the past 2 weeks have you had bloating (sensation of too much gas in the abdomen)?   all of the time, most of the time, some of the time, a little of the time, never |
| 1. How often during the past 2 weeks have you been troubled by excessive passage of gas through the anus?   all of the time, most of the time, some of the time, a little of the time, never |
| 1. How often during the past 2 weeks have you been troubled by strong burping or belching?   all of the time, most of the time, some of the time, a little of the time, never |
| 1. How often during the past 2 weeks have you been troubled by gurgling noises from the abdomen?   all of the time, most of the time, some of the time, a little of the time, never |
| 1. How often during the past 2 weeks have you been troubled by frequent bowel movements?   all of the time, most of the time, some of the time, a little of the time, never |
| 1. How often during the past 2 weeks have you found eating to be a pleasure?   all of the time, most of the time, some of the time, a little of the time, never |
| 1. Because of your illness, to what extent have you restricted the kinds of food you eat?   very much, much, somewhat, a little, not at all |
| 1. During the past 2 weeks, how well have you been able to cope with everyday stresses?   extremely poorly, poorly, moderately, well, extremely well |
| 1. How often during the past 2 weeks have you been sad about being ill?   all of the time, most of the time, some of the time, a little of the time, never |
| 1. How often during the past 2 weeks have you been nervous or anxious about your illness?   all of the time, most of the time, some of the time, a little of the time, never |
| 1. How often during the past 2 weeks have you been happy with life in general?   never, a little of the time, some of the time, most of the time, all of the time |
| 1. How often during the past 2 weeks have you been frustrated about your illness?   all of the time, most of the time, some of the time, a little of the time, never |
| 1. How often during the past 2 weeks have you been tired or fatigued?   all of the time, most of the time, some of the time, a little of the time, never |
| 1. How often during the past 2 weeks have you felt unwell?   all of the time, most of the time, some of the time, a little of the time, never |
| 1. Over the past week, have you woken up in the night?   every night, 5-6 nights, 3-4 nights, 1-2 nights, never |
| 1. Since becoming ill, have you been troubled by changes in your appearance?   a great deal, a moderate amount, somewhat, a little bit, not at all |
| 1. Because of your illness, how much physical strength have you lost?   a great deal, a moderate amount, some, a little bit, none |
| 1. Because of your illness, to what extent have you lost your endurance?   a great deal, a moderate amount, somewhat, a little bit, not at all |
| 1. Because of your illness, to what extent do you feel unfit?   extremely unfit, moderately unfit, somewhat unfit, a little unfit, fit |
| 1. During the past 2 weeks, how often have you been able to complete your normal daily activities (school, work, household)?   all of the time, most of the time, some of the time, a little of the time, never |
| 1. During the past 2 weeks, how often have you been able to take part in your usual patterns of leisure or recreational activities?   all of the time, most of the time, some of the time, a little of the time, never |
| 1. During the past 2 weeks, how much have you been troubled by the medical tI;eatment of your illness?   very much, much, somewhat, a little, not at all |
| 1. To what extent have your personal relations with people close to you (family or friends) worsened because of your illness?   very much, much, somewhat, a little, not at all |
| 1. To what extent has your sexual life been impaired (harmed) because of your illness?   very much, much, somewhat, a little, not at all |
| 1. How often during the past 2 week, have you been troubled by fluid or food coming up into your mouth (regurgitation)?   all of the time, most of the time, some of the time, a little of the time, never |
| 1. How often during the past 2 weeks have you felt uncomfortable because of your slow speed of eating?   all of the time, most of the time, some of the time, a little of the time, never |
| 1. How often during the past 2 weeks have you had trouble swallowing your food?   all of the time, most of the time, some of the time, a little of the time, never |
| 1. How often during the past 2 weeks have you been troubled by urgent bowel movements?   all of the time, most of the time, some of the time, a little of the time, never |
| 1. How often during the past 2 weeks have you been troubled by diarrhoea?   all of the time, most of the time, some of the time, a little of the time, never |
| 1. How often during the past 2 weeks have you been troubled by constipation?   all of the time, most of the time, some of the time, a little of the time, never |
| 1. How often during the past 2 weeks have you been troubled by nausea?   all of the time, most of the time, some of the time, a little of the time, never |
| 1. How often during the past 2 weeks have you been troubled by blood in the stool?   all of the time, most of the time, some of the time, a little of the time, never |
| 1. How often during the past 2 weeks have you been troubled by heartburn?   all of the time, most of the time, some of the time, a little of the time, never |
| 1. How often during the past 2 weeks have you been troubled by uncontrolled stools?   all of the time, most of the time, some of the time, a little of the time, never |

Calculation of the score: Each subscore ranges from 0 to 4 points. The maximum Gastrointestinal
Quality of Life Index score is 144 points, with higher scores indicating better function.

most desirable option: 4 points

least desirable option: 0 points

GIQLI score: sum of the points
